# Supplementary material for: Discrimination of the hierarchical structure of cortical layers in 2-photon microscopy data by combined unsupervised and supervised machine learning
Source: Sci Rep. 2019 May 15;9:7424. doi: 10.1038/s41598-019-43432-y (PMC6520410; doi:10.1038/s41598-019-43432-y)
Supplement: Supplementary file 1 — SUPPLEMENTARY INFO - Discrimination of the hierarchical structure of cortical layers in 2-photon microscopy data by combined unsupervised and supervised machine learning [file 41598_2019_43432_MOESM1_ESM.pdf]

Supplementary Information

Discrimination of the hierarchical structure of cortical layers in 2-photon microscopy data by combined unsupervised and supervised machine learning

Dong Li[1,*], **Melissa Zavaglia**[1,5,*], **Guangyu Wang**[2,3*], **Hong Xie**[4], **Yi Hu**[3,2], **Rene Werner**[1], **Ji-Song Guan**[2,3], **Claus C. Hilgetag**[1,6,#]

[1] Institute of Computational Neuroscience, University Medical Center Hamburg-Eppendorf, Hamburg, 20246, Germany
[2] School of Life Science and Technology, ShanghaiTech University, Shanghai, 201210, China
[3] School of Life Sciences, Tsinghua University, Beijing, 100086, China
[4] Institute of Brain-Intelligence Technology, Zhangjiang Laboratory, Shanghai Research Center for Brain Science and Brain-Inspired Intelligence, 200031, China
[5] Focus Area Health, Jacobs University Bremen, Bremen, 28759, Germany
[6] Department of Health Sciences, Boston University, Boston, MA 02215, USA

* These authors contributed equally to this work

Corresponding author (#):

Prof. Claus C. Hilgetag, PhD

Professor & Director, Institute of Computational Neuroscience,
University Medical Center Eppendorf, Martinistraße 52, 20251 Hamburg, Germany

Website: www.uke.de/icns,
E-mail: c.hilgetag@uke.de

Table S1. Refined maps between reference c-Layers from SSp areas and the manually labelled layers. Similar to the VISp areas, some layers were merged after visual inspection.

Reference location name	Reference c-Layer structure	Manually labelled layer structure
M23R4	[1], [2], [3], [4, 5]	[1], [2], [3], [5]
M248L5	[1], [2], [3], [4, 5]	[1], [2], [3], [5]
M226R5	[1, 2, 3], [4], [5]	[1, 2, 3], [4], [5]
M337R6	[1], [2], [3], [4], [5]	[1], [2], [3], [4], [5]
M16R4	[1], [2], [3], [4], [5]	[1], [2], [3], [4], [5]
M262L4	[1], [2], [3], [4, 5]	[1], [2], [3], [5]
M261R4	[1], [2], [3], [4], [5]	[1], [2], [3], [4], [5]

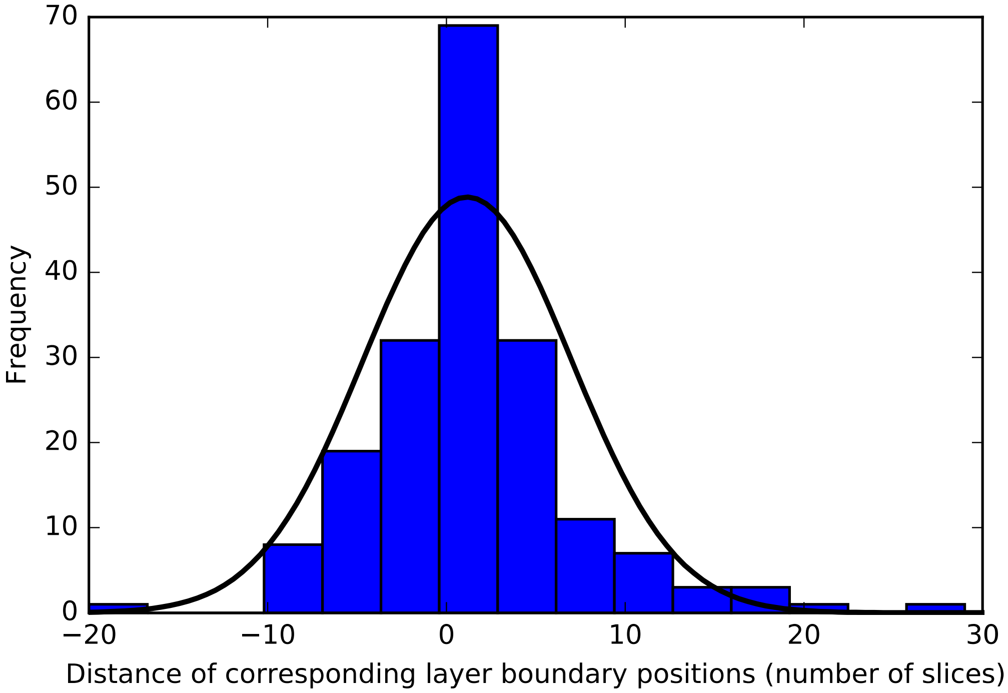

Figure S1. Distribution of the differences of layer boundary positions as manually determined by comparison of corresponding data of the two expert raters. The black line indicates a Gaussian distribution fit to the data (mean value μ = 1.17; standard deviation σ = 5.85; related uncertainty, that means, standard deviation of pairs of measurements $1/\sqrt{2}\ \sigma$ = 4.13).

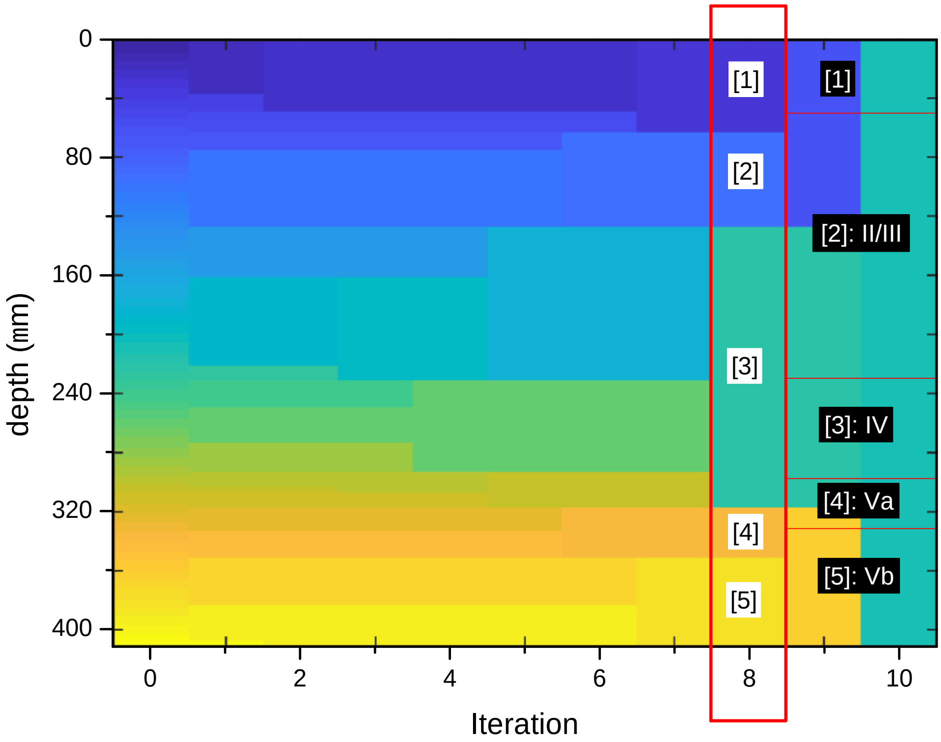

Figure S2. Comparison between unsupervised clustering results of M248R3 with feature set F1 and its corresponding manually labelled layers. The red rectangle highlights the reference c-layers we used (layer names indicated by black text with white background). To its right side, the red horizontal lines indicate the B-Layer boundaries (layer names by white text and black background).

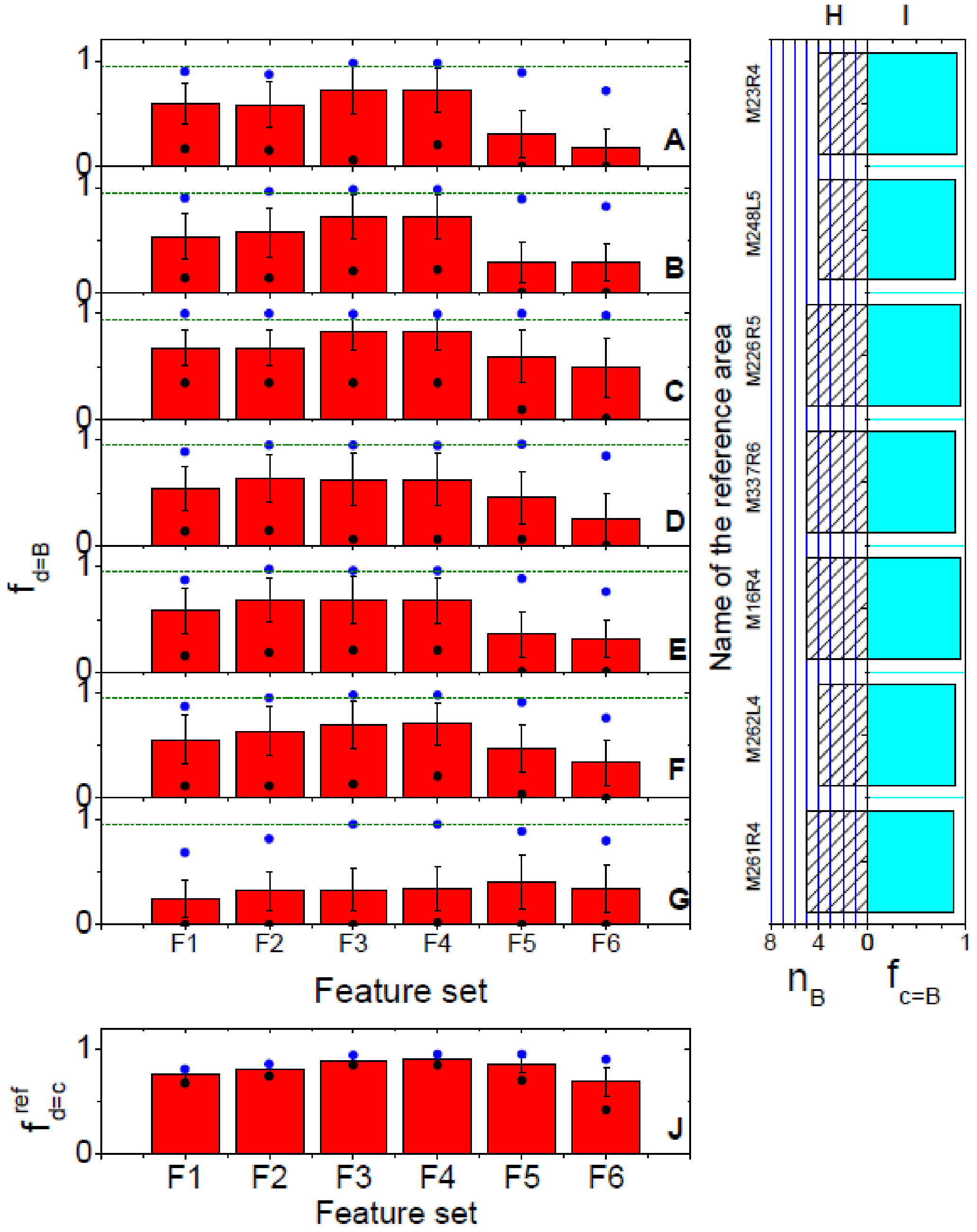

Figure S3. Supervised learning part evaluations for SSp reference c-Layers. All figure details and symbol meanings similar to manusript Figure 7.

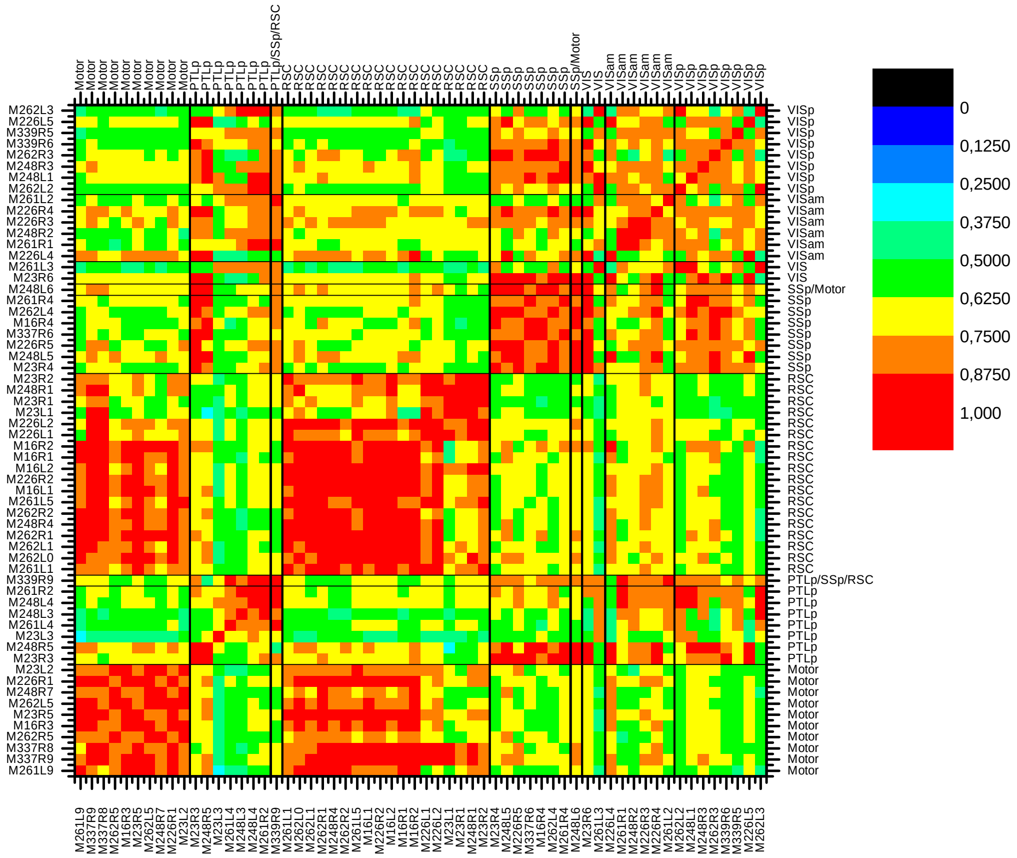

Figure S4. Similarity of first manual labels for any pair of locations.

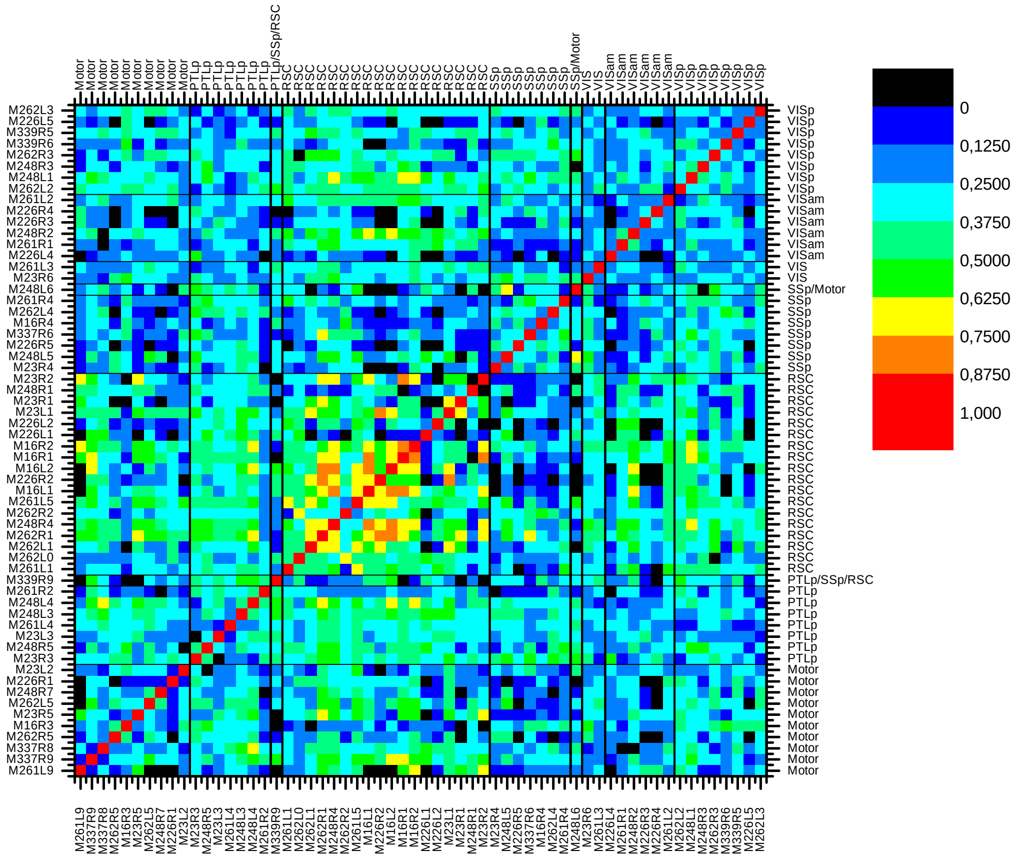

Figure S5. Similarity of hierarchical clustering results for any pair of datasets and using feature set F1 for clustering.

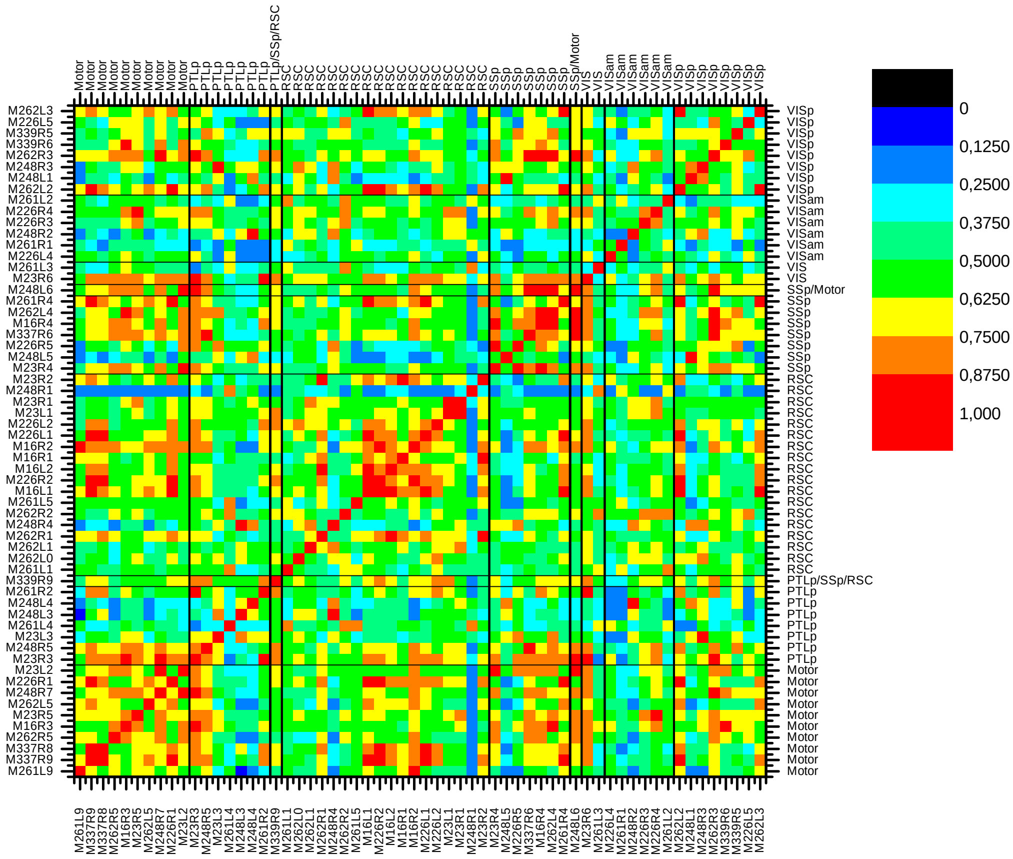

Figure S6. Similarity of layer discrimination results for any pair of dataset locations, using feature set F3 for supervised learning and taking M226R3 as reference location.

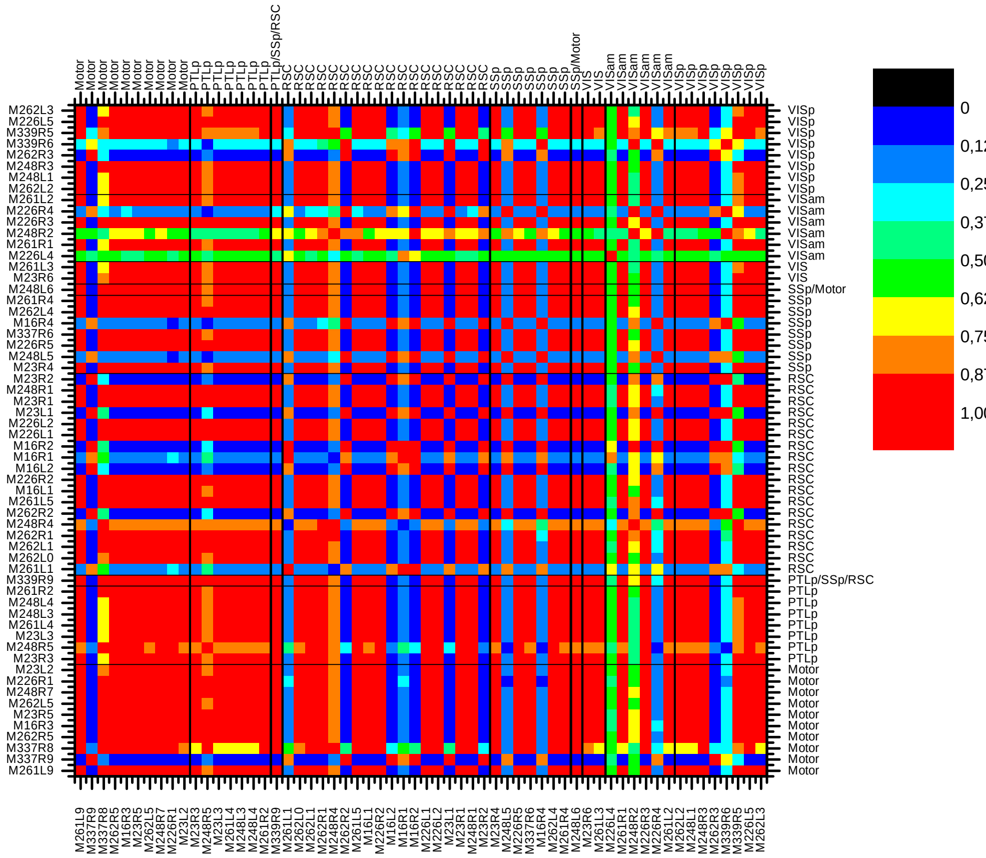

Figure S7. Similarity of discrimination results for any pair of dataset locations, using feature set F6 for supervised learning and taking M16R4 as reference location.
